# Genomic diversity and adaptation of *Salmonella enterica* serovar Typhimurium from analysis of six genomes of different phage types

**DOI:** 10.1186/1471-2164-14-718

**Published:** 2013-10-20

**Authors:** Stanley Pang, Sophie Octavia, Lu Feng, Bin Liu, Peter R Reeves, Ruiting Lan, Lei Wang

**Affiliations:** 1 School of Biotechnology and Biomolecular Sciences, University of New South Wales, Sydney, New South Wales 2052, Australia; 2 School of Molecular Bioscience, University of Sydney, Sydney, New South Wales 2006, Australia; 3 TEDA School of Biological Sciences and Biotechnology, Nankai University, Tianjin, China

**Keywords:** Typhimurium, Genome, Next generation sequencing, Phage type, Evolution, Single nucleotide polymorphism

## Abstract

**Background:**

*Salmonella enterica* serovar Typhimurium (or simply Typhimurium) is the most common serovar in both human infections and farm animals in Australia and many other countries. Typhimurium is a broad host range serovar but has also evolved into host-adapted variants (i.e. isolated from a particular host such as pigeons). Six Typhimurium strains of different phage types (defined by patterns of susceptibility to lysis by a set of bacteriophages) were analysed using Illumina high-throughput genome sequencing.

**Results:**

Variations between strains were mainly due to single nucleotide polymorphisms (SNPs) with an average of 611 SNPs per strain, ranging from 391 SNPs to 922 SNPs. There were seven insertions/deletions (indels) involving whole or partial gene deletions, four inactivation events due to IS*200* insertion and 15 pseudogenes due to early termination. Four of these inactivated or deleted genes may be virulence related. Nine prophage or prophage remnants were identified in the six strains. Gifsy-1, Gifsy-2 and the *sopE2* and *sspH2* phage remnants were present in all six genomes while Fels-1, Fels-2, ST64B, ST104 and CP4-57 were variably present. Four strains carried the 90-kb plasmid pSLT which contains several known virulence genes. However, two strains were found to lack the plasmid. In addition, one strain had a novel plasmid similar to Typhi strain CT18 plasmid pHCM2.

**Conclusion:**

The genome data suggest that variations between strains were mainly due to accumulation of SNPs, some of which resulted in gene inactivation. Unique genetic elements that were common between host-adapted phage types were not found. This study advanced our understanding on the evolution and adaptation of Typhimurium at genomic level.

## Background

*Salmonella enterica* serovar Typhimurium is one of the leading causes of *Salmonella*-related gastroenteritis in humans. The Anderson phage typing scheme [1], in which Typhimurium is divided into subtypes based on phenotypic variation, resulted from the susceptibility or resistance to a set of bacteriophages, has been used for epidemiological typing for the past 40 years. The success of phage typing has been well documented in the tracking of epidemiological phage types such as DT204 in the early 1970s, and recently, the epidemic strain DT104 causing outbreaks worldwide [2]. Typhimurium is a broad host range serovar but has also evolved into host-adapted variants with some phage types being more commonly isolated from particular hosts. For example, DT2 is commonly isolated from pigeons and is associated with systemic disease in pigeons [3]. Host adaptation ensures the circulation within an animal population, and this process may have evolved through the acquisition of virulence determinants and/or loss of gene functions [4].

In Australia, three phage types were found to be predominantly isolated from human infections as well as in animals based on surveillance data from 1996 to 2011 [5]. DT135 has been most prevalent, causing 20-27% of Typhimurium infections in the past 10 years, and is clearly established in Australia as an endemic phage type infecting humans. DT9 is less frequent than DT135 in human infections but is the most frequent phage type in farm animals, with almost twice the frequency. DT170/108 has been increasing steadily over recent years and became the most frequent phage type in 2004, surpassing DT135 [5].

We previously addressed the origins and relationships of the common phage types in Australia using single nucleotide polymorphisms (SNPs) as molecular markers [6]. SNPs discovered by amplified fragment length polymorphism analysis were used to determine the genetic relationship of 46 Typhimurium isolates from nine phage types [7]. SNP typing was later extended in our recent study to incorporate additional SNPs obtained from comparison of five Typhimurium genomes (DT2, LT2, SL1344, D23580 and NCTC13348) [6]. A total of 44 SNPs were able to resolve 221 Typhimurium isolates with 45 phage types into four major clusters (SNP clusters I to IV). However, the SNPs used clearly still have limited discriminatory power. There were SNP profiles which contained many different phage types. Phage types that are prevalent in Australia, including DT9, DT135 and DT197, were clustered with other phage types. Due to SNP discovery bias [8], more SNPs from strains representing the diversity within the SNP profiles or phage types will be required to increase the resolution of SNP based typing.

Genome variations have been observed in studies comparing whole genome sequences of Typhimurium strains LT2 (DT4), SL1344 (DT44), NCTC13348 (DT104), and D23480 (unknown phage type), with strain specific pseudogenes and SNPs found within each genome [9]. Mobile elements such as prophages, transposons, plasmids and insertions sequences may also vary among Typhimurium genomes. The aims of this study were to use comparative genomics to identify the genetic diversity between multiple prevalent phage types and try to begin to elucidate the genetic basis for the predominance of certain phage types and host adaptation.

## Methods

### Bacterial strains and genomic DNA isolation

Six strains were selected to represent the spectrum of Typhimurium diversity (Table 1) based on a previous SNP typing study using 44 SNP markers [7]. The selection criteria were based on SNP profiles as well as the prevalence of certain phage types from epidemiology data collected from the National Enteric Pathogen Surveillance Service (NEPSS) [5,10]. A DT99 isolate (host-adapted to pigeons) was selected for comparison against other Typhimurium isolates to identify potential genetic factors involved in host adaptation. Genomic DNA from each strain was extracted using the phenol/chloroform method as described previously [11].

**Table 1:** Strains sequenced in this study

**Strain name**	**Phage type**	SNP profile*	**Source**	**Location**	**Year of isolation**
L796	DT99	10	Avian	UK	2002
L847	DT197	30	Human	Australia	2003
L852	DT135a	33	Human	Australia	2008
L904	DT9	7	Human	Australia	1997
L927	DT12a	21	Human	Australia	1997
L945	DT108	1	Avian	Australia	1997

*SNP profile that was defined in Pang *et al. *[6].

### DNA sequencing and assembly

DNA libraries were prepared with insert size of 500 bp and were sequenced using the Illumina Genome Analyzer (Illumina). We used 2 × 50 bp paired end sequencing. Contigs were assembled using the Short Oligonucleotide Analysis package (SOAP) (version 1.04) [12]. SOAPdenovo settings were set with the following parameters: K value = 31, –d = 1 and D = 1 to generate scaffolds. A K value of 31 gave the best N50 contig size. Large scaffolds and short contigs generated by SOAPdenovo were aligned to the Typhimurium LT2 genome (NC_003197) using progressiveMauve [13].

Accession numbers for the genome sequences obtained in this study were AROB00000000-AROG00000000.

### Identification of SNPs

Mapping of reads against the Typhimurium LT2 genome was performed using the Burrows-Wheeler alignment (BWA) tool (version 0.5.8) [14]. The output generated lists including the number of Illumina reads covering each nucleotide position, which corresponds to the reference genome. A custom script was used to extract SNPs according to the position on the reference genome and the number of reads covering the region containing the SNP. Some SNPs could result from errors in mapping or sequencing. Therefore, further filtering was performed.

A cutoff of 20 reads covering the SNP site was used initially to remove SNP sites with low coverage. We also used SOAPdenovo to assemble reads into contigs and then compared with the LT2 genome to identify SNPs. *de novo* assembly was done using quality trimmed reads. This may have reduced the number of SNPs. *de novo* assembly eliminated the problem with reads that may be mapped to spurious positions (mostly repeats or homologous regions) with mismatches being called SNPs. For SOAPdenovo assembly, reads were trimmed after the first base falling below Q7. The read was only excluded if the length of reads was 17 bases after the trimming. For BWA mapping, no filtering of reads was performed.

SNPs identified by both methods were compared. These common SNPs were manually inspected using SAMtools (version 0.1.7) [15] and its in-built function, Tview, for visualising the mapping of reads at each SNP position. SNPs identified from BWA mapping were further filtered using SAMtools by SNP quality. Any SNPs with quality score of less than 20 were removed.

SAMtools were used to manually confirm all SNPs for our initial analysis of one genome. We found a consistent pattern where SNPs were in fact sequencing errors when the region was covered only by ends of reads which is known to have poorer quality. For SNP sites with heterogeneous reads (i.e. at least two bases were called at the same site from different reads), the majority of the SNPs were genuine if the SNP was supported by ≥70% of the reads. A small proportion of SNP calls were genuine for those falling between 30% and 70%. None of the SNPs was genuine if less than 30% of reads supported the SNP. In case we removed genuine SNPs of lower than 20X coverage, we inspected SNP sites between >10 and <20 reads coverage and rescued genuine SNPs and added to the final set of SNPs. These genuine SNPs with lower than 20X coverage generally had 100% support for a SNP. Non-genuine SNPs were typically located at the ends of the reads and visual inspection identified them with relatively low subjectivity.

Another custom script was used to determine whether SNPs were synonymous (sSNP) or non synonymous (nsSNP). The validated SNPs were also used for comparison to other Typhimurium genome sequences D23580 (Accession No.: FN424405) [9], 14028 S (DT133) (Accession No.: CP001363) [16], T000240 (DT12) (Accession No.: AP011957) [17], NCTC13348 (DT104) (Accession No.: XB000031) [18] and SL1344 (DT44) (Accession No.: FQ312003) [19]. Additionally, an unpublished genome sequence of an unnamed DT2 pigeon isolate (http://www.sanger.ac.uk) was included for comparison. SNPs were then used to generate a maximum parsimony (MP) tree using the PAUP package [20] to illustrate the genetic relationships of Typhimurium isolates. *S. enterica* serovar Enteritidis strain PT4 (NCTC13349) (Accession No.: AM933172) and *S. enterica* serovar Choleraesuis strain SC-B67 (NC_006905) (Accession No.: NC_006905) were used as outgroups.

### Distribution of insertions and deletions

Insertions and deletions (indels) were identified using the mapping data from BWA [14]. The distance between the paired ends of a read were first calculated by mapping them to the reference genome. Any pairs with distance larger than the average insert size of 500 bp potentially contain a deletion in the newly sequenced genome. There were at least 10 fragments (paired end reads) to identify a deletion. The regions containing the potential deletion were examined using the Tview function in SAMtools [15] to locate the approximate breakpoint of the deletion and determine the number of reads covering the region up to the breakpoint with at least 20X coverage for the confirmed deletions. It should be noted this approach cannot identify small indels. We only looked for deletions of at least 500 bp in the new genome. This approach cannot identify large insertions in the new genome either. Potential indel events were further compared to other publicly available genomes that were found to be closely related from the SNP-based phylogeny to determine whether they were present in the other genomes [6].

Identification of new IS insertions were done using a similar method. Paired end reads with one end mapping to an existing IS location in the reference genome while the other end mapping to a distance location. The insertion point was determined visually based on the typical pattern of abrupt end of reads mapping as no overlapping reads can be found at the insertion point. We did not determine the precise location of the insertion using reads that contain part of unique sequence and part of IS sequence.

### Identification and annotation of unique sequences

Using progressiveMauve [13], some contigs were not able to be aligned. These unaligned sequences were re-aligned using BLASTn against reference Typhimurium genome strain LT2 to confirm whether they were duplicated sequences or unique regions. After duplicated sequences were identified and removed, contigs that did not belong to LT2 were compared again using BLASTn against the GenBank non-redundant nucleotide collection database to determine their homologues.

### Phylogenetic analysis

Maximum parsimony was done using PAUP [21] with heuristic search based on tree bisection and reconnection (TBR) swap method. *S. enterica* serovars Enteritidis and Choleraesuis strains were used as an outgroup. Outgroup genomes were aligned using progressiveMauve to the LT2 reference genome. A custom script was used to extract the nucleotide for each outgroup genome at the corresponding SNP containing locations.

## Results and discussion

### Selection of strains and genome sequencing

Two isolates were selected from SNP cluster I from our previous study [6], including L945 (DT108) and L927 (DT12a). L945 is a DT108 isolate but in Australia, DT108 is also known as DT170. This phage type contributes to approximately 15% of Typhimurium infections in Australia [5]. The phage type DT12a was a prevalent phage type in Australia but has decreased in recent years [5]. However, DT12a was reported with increasing infection during poultry surveillance in the neighbouring New Zealand [22] and multidrug resistance as reported by Lawson *et al.*[23]. L847 (DT197) was selected to represent SNP cluster II and is one of the most prevalent phage types [24]. There were three strains selected for SNP cluster III, L852 (DT135a), L904 (DT9) and L796 (DT99). DT135 and DT9 are the two most prevalent phage types in Australia. L852 is a DT135a strain, a variant of DT135 which belongs to the same SNP profile as other DT135 strains. A DT135a strain was selected over DT135 since it has been increasing in frequency in recent years in Australia. L796, a DT99 isolate, was selected as a representative of host adapted phage type. DT99 has been commonly isolated from pigeons [25]. The genome data of this strain provides a comparison with DT2, a phage type adapted to pigeons which is currently being sequenced by the Wellcome Trust Sanger Institute (http://www.sanger.ac.uk/resources/downloads/bacteria/salmonella.html). All except DT99 selected in this study are broad host range phage types.

The average number of reads (50 bp) generated per genome was ~9,200,000 and the coverage depth on average for all genomes was ~75X, with the lowest coverage of 63X (Table 1). Coverage of the reads against the LT2 reference genome ranged from 1 to 662 reads per site. Those with low coverage are most likely reads with multiple sequencing errors aligned in the wrong position. Genome coverage based on the LT2 reference was approximately 95% on average for the six genomes, with 96% coverage for strains L796 (DT99), L847 (DT197), L852 (DT135a) and L904 (DT9), 91% for L945 (DT108) and 98% for L927 (DT12a). The difference in coverage was likely due to genome sequences present in the LT2 reference but absent from the strains sequenced. Mapping was also performed on the plasmid pSLT associated with the LT2 reference, which is described in detail below.

### Single nucleotide polymorphisms in Typhimurium strains

We used two approaches to identify SNPs: mapping to the reference genome and *de novo* assembly. We first used BWA [14] to map reads against the LT2 genome which generated a large set of possible SNP sites (Table 2). For each potential SNP, a cutoff of 20 reads covering the SNP was required in order to exclude SNPs generated from incorrect mapping or sequencing errors. After filtering the SNP sites with low coverage, the average number of SNPs for the six strains was 2,631 SNPs. Strain L796 (DT99) contained the most number of SNPs with 3,323 SNPs identified. We then used SOAPdenovo to assemble reads into contigs and then compared with the LT2 genome to identify SNPs. Four genomes, L796 (DT99), L847 (DT197), L945 (DT108) and L927 (DT12a) had lower numbers of SNPs than direct mapping of reads using BWA. SNPs identified by both methods were further filtered by SNP quality and SNPs with a quality score of less than 20 were also removed to derive a final set of SNPs. There were on average 611 SNPs, ranging from 391 SNPs in L945 (DT108) to 922 SNPs in L796 (DT99). The confirmed number of SNPs was approximately a quarter of the original number of SNPs identified and this was due to mismatched reads alignments and sequencing errors.

**Table 2:** **General features of the six *S. enterica *serovar Typhimurium genomes sequenced in this study**

**Strain (phage type)**	**L796 (DT99)**	**L847 (DT197)**	**L852 (DT135a)**	**L904 (DT9)**	**L927 (DT12a)**	**L945 (DT108)**
Total no. of reads	8,204,694	13,168,608	8,746,596	8,844,626	7,363,632	8,894,448
Total sequences (bp)	434,848,782	697,936,224	463,569,588	468,765,178	390,272,496	471,405,744
Coverage depth average	69	121.1	62.7	63.6	62.9	73.2
Coverage depth range	1-541	1-581	1-662	1-628	1-477	1-594
Percentage match to LT2 chromosome*	96	96	96	96	98	91
Percentage match to LT2 pSLT*	96	96	86	90	0.8	2.6
SNP sites (by BWA)	3,323	2,268	2,615	2,545	2,616	2,422
SNP sites (by SOAP/progressiveMauve)	2,477	1,349	2,862	3,089	1,349	1,866
No. and % of nSNP^#^	391 (42.4)	261 (42.4)	224 (42.1)	311 (46.8)	250 (46.1)	170 (43.5)
No. and % of sSNP^#^	327 (35.5)	232 (37.7)	200 (37.6)	216 (32.5)	200 (36.9)	145 (37.1)
No. and % of Intergenic SNPs^#^	172 (18.7)	108 (17.6)	95 (17.9)	123 (18.5)	84 (15.5)	67 (17.1)
No. and % of single base deletions^#^	32 (3.5)	14 (2.3)	13 (2.4)	14 (2.1)	8 (1.5)	9 (2.3)
Total SNPs	922	615	532	664	542	391

*Percentages of the LT2 chromosome and LT2 plasmid pSLT were covered by reads from each sequenced genome.

^#^In brackets are percentages. nSNP and sSNP are nonsynonymous and synonymous SNPs, respectively.

The SNPs were classified into four categories: non-synonymous (nsSNP), synonymous (sSNP), intergenic (IG) and single base indels. IG SNPs on average accounted for approximately 17.5% of the total number of SNPs in each strain. The average percentage of sSNP for each strain was 36.2% with L904 (DT9) having the lowest ratio of sSNPs (32.5%) while the nsSNPs ranged from 42.1% to 46.8% with an average of 43.9%. Single base indels accounted for only 2% of the SNPs. L796 (DT99) had the highest number of indels (32 indels) representing 3.5% of SNPs, while L927 (DT12a) had the lowest with eight indels which accounted for 1.5% of all SNPs. The ratio between sSNPs and nsSNPs ranged from 0.70 to 0.89.

### Genome tree based on maximum parsimony analysis

**Figure 1:**
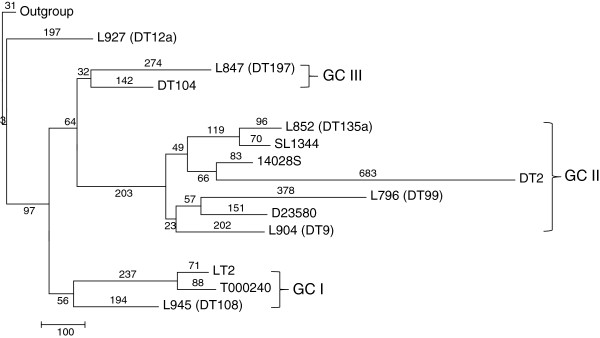
**The maximum parsimony tree of 13** ***S. enterica *serovar Typhimurium genomes based on genome SNPs.** The number on the internal and terminal branches corresponds to the number of SNPs supporting each corresponding branch. Note that SNPs for strains L796 (DT99), L847 (DT197), L852 (DT135a), L904 (DT9), L927 (DT12a) and L945 (DT108) were obtained from genome sequencing in this study, while others were from publicly available genomes. *S. enterica* serovars Enteritidis and Choleraesuis were used as the outgroup. Genome clusters (GC) are demarcated by brackets and identified with roman numerals. Phage types DT2 and DT99 are host-adapted (pigeons).

The SNP data from the six isolates were compared with those from seven publicly available Typhimurium genomes: LT2, SL1344, D23580, 14028 S, T000240 and DT104 and an incomplete DT2 genome. A maximum parsimony (MP) tree (Figure 1) was constructed using 3,368 SNPs with two other serovars, Choleraesuis strain SC-B67 and Enteritidis PT4 strain NCTC 13349, as the outgroup. Only one MP tree was generated. The homoplasy index (HI) was 0.112, indicating the presence of parallel or reverse changes as discussed below. The HI was comparable to what was found previously using SNP typing (HI = 0.17). SNPs identified from prophages were not used to generate the MP tree as the addition of prophage SNPs resulted in a HI of 0.212. External branches contained more SNPs than internal branches.

In the MP tree, the Typhimurium strains were distributed into three clusters designated as genome cluster (GC) I, GC II and GC III. Strain L927 (DT12a) was found to have diverged the earliest since it was closest to the outgroup and shared most recent common ancestor with the three clusters which were grouped together and supported by 97 SNPs. GC II and GC III were grouped together and supported by 64 SNPs. GC I contained three isolates L945 (DT108), T000240 and LT2 and was supported by 56 SNPs. GC II contained four publicly available genomes DT2, 14028 S, SL1344, D23580, and three NGS strains; L852 (DT135a), L904 (DT9) and L796 (DT99). GC II was well separated from GC III and was supported by 203 SNPs. GC III contained two isolates, DT104 and L847 (DT197) and was supported by 32 SNPs. The strain specific SNPs for L796 (DT99), L847 (DT197), L852 (DT135a), L904 (DT9), L927 (DT12a) and L945 (DT108) were 378, 274, 96, 202, 197 and 194, respectively. Amongst the publicly available genomes, DT2 had a large number of strain specific SNPs (683 SNPs) while the strain specific SNPs for the remaining six genomes (LT2, T000240, SL1344, 14028S, DT104 and D23580) ranged from 71 to 151.

In this study, the HI was greater than 0 which suggests that some SNPs had conflicting phylogenetic signals. The SNPs were mapped onto the internal and external branches in the MP tree. There were 33 SNPs present in multiple internal branches, indicating reverse/parallel changes which were likely to be resulted from recombination between lineages. Of these, 28 were IG SNPs. For the external branches, the distribution of SNPs was used as an indicator of recombination within a lineage. It has been previously suggested that recombination event constitutes the presence of three or more substitution events within a 1 kb region [26]. There were 47, 31, 30, 10, 14 and 18 SNPs, which resulted in 34, 17, 24, 6, 10 and 13 potential recombinational segments in strains L796, L904, L847, L852, L927 and L945, respectively. The recombination to mutation rate was similar in L796, L847, L904 and L945 with approximately 5% of their SNPs resulting from recombinational events while L852 had the lowest with only 1.9%. Despite the presence of parallel/reverse mutations, the resulting phylogeny was generally consistent with our previous SNP typing study. This suggests that the extent of recombination has not yet distorted the evolutionary relationships among the strains and may not play a major role in driving the genetic diversity within this serovar.

### Insertion sequences

Insertion sequences (IS) play an important role in bacterial evolution as transposition can potentially inactivate a gene [27]. *S. enterica* is known to carry three insertion sequences, IS*1,* IS*3* and IS*200*[28]. IS*1* is rarely detected in Typhimurium [28] and was not found among the six NGS strains. IS*3* was previously found in a high proportion of Typhimurium isolates included in *Salmonella* reference collection A (SARA) [29]. However, only a single copy of IS*3* sequence was found at the same location in the six strains sequenced. IS*200* was the only IS commonly found in the genome strains.

**Figure 2:**
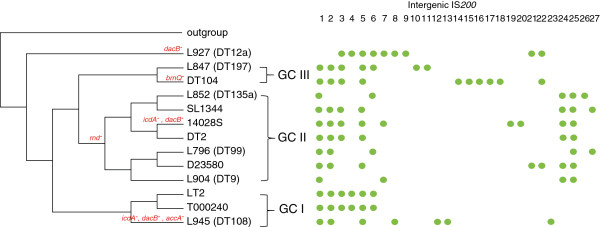
**The distribution of IS*200 *in *S. enterica *serovar Typhimurium genomes.** The IS*200* insertions in intergenic regions with copies numbered from IS*200*_1 to IS*200*_27 are displayed on the right. IS*200* causing truncations of genes are labeled on the affecting nodes with the gene symbols. The maximum parsimony tree is the same tree as Figure 1 but only displays the topology and the branch lengths are not proportional to the number of SNPs. The genome clusters (GC) are marked by roman numerals.

There were 32 different IS*200* locations found among the 13 Typhimurium genomes, with 27 located in IG regions (Figure 2) and five in genic regions. The genic insertions are described in the gene disrupting events section below. Several of the IG IS*200* locations were found in the same location in multiple genomes. The reference genome LT2 contained six copies of IS*200* in IG regions, which were designated as IS*200*_1 – IS*200*_6. The other IS*200* copies are assigned as IS*200*_7 to IS*200*_27. Fifteen of these IS*200* locations (IS*200*_9 – IS*200*_20, IS*200*_23 and IS*200*_26) occurred only in a single strain as shown in Figure 2. The average number of copies of IG *IS200* was seven ranging from four to 10 copies. IS*200*_1 and IS*200*_2 were likely to have been gained by the most recent common ancestor of GC I, GC II and GC III since L927 (DT12a) does not contain either IS. Additionally, IS*200*_2 was likely to have been lost by L904 (DT9) and L852 (DT135a). Similarly, IS*200*_24 and IS*200*_25 were likely to have been gained by the most recent common ancestor of GC II as all except one strain contained IS*200*_24 and all contained IS*200*_25 (Figure 2).

### Gene disruption events

Five IS insertions were found within a gene. The IS*200* insertion at the *accA* gene (L945 (DT108)) and *brnQ* (DT104) occurred in a single strain. The insertions at *dacB*, *icdA* and *rnd* were shared amongst two or more strains. The disruption of *rnd* appeared to have occurred early during the divergence of GC II as all strains belonging to this cluster had the IS*200* insertions. The insertions of IS*200* into *dacB* and *icdA* occurred three and two times respectively. It appeared to be independent for both cases as the gene disruptions were present in strains from different clusters (Figure 2). We did not determine whether the independent insertions occurred at the exact same site for each gene as potential hotspots for IS insertion.

The insertion into *icdA*, found in L945 and 14028 S, may have an advantageous effect, with *icd* mutants known to resist low levels of nalidixic acid [30]. The non-functioning *icd* gene results in the lack of citrate synthase activity, allowing the accumulation of citrate which has an unexplained effect on nalidixic acid resistance.

*AccA* codes for carboxyltransferase subunit and can be inhibited by pseudopeptide pyrrolidinedione antibiotics such as moiramide B. Pyrrolidinedione resistant strains of *E. coli* and *Staphylococcus aureus* have been found to contain mutations in the subunits of AccA [31]. The IS*200* disruption of *accA* in L945 (DT108) may be related to antimicrobial resistance.

Another inactivated gene *dacB* was found in strains L945 (DT108) and L847 (DT197). This gene encodes a penicillin-binding protein 4 (PBP4) which functions as a trap target for β-lactams [32]. Interestingly, the inactivation of nonessential *dacB*-coded PBP4 triggers overproduction of β-lactamase AmpC and the specific activation of the CreBC (BlrAB) two-component regulator leading to a high level of β-lactam resistance [33].

### Prophage insertions and deletions

Typhimurium strains harbor many prophages [34-40]. The pairwise alignments of the NGS strains against the reference LT2 genome were used to determine the presence and absence of prophages. In total, nine prophages or prophage remnants including Fels-1, Fels-2, Gifsy-1, Gifsy-2, Gifsy-3, ST104, CP4-57, ST64B and SopEΦ were found in the six NGS genomes (Table 3).

**Table 3:** Distribution of prophages in the genomes sequenced in this study

**Prophage**	**Virulence genes**	**Function**	**Strain (phage type)**					
			**L796 (DT99)**	**L847 (DT197)**	**L852 (DT135a)**	**L904 (DT9)**	**L945 (DT108)**	**L927 (DT12a)**
Fels-1	*nanH*	Neuraminidase	-	+	-	-	-	-
	*sodCIII*	Putative Cu/Zn superoxide dismutase						
Gifsy-1	*gogA*	Similar to *pipA* in SPI5	+	+	+	+	+	+
	*gipA*	Effector proteins for enhanced growth in Peyer’s patches						
	*gogB*	Similar to type III secretion protein						
	*gogD*	Similar to *pagK*						
sopE2*	*pagK, mig-3, pagO, sopE2loci*	Invasion-associated effectors that activate different sets of RhoGTPases of the host cell	+	+	+	+	+	+
sspH2*	*sspH2*	SPI-2 type III secretion system	+	+	+	+	+	+
Gifsy-2	*sodCI*	Periplasmic Cu/Zn superoxide dismutase	+	+	+	+	+	+
	*sopE*	SPI-1-dependent translocated effector protein						
	*gtgE*	Cytoplasmic protein						
	*sseI*	Secreted effector protein						
	*gtgF*	Similar to macrophage survival gene (*msgA*)						
	*grvA*	Antivirulence gene						
ST64B	*sseK*	Type III secreted effector protein	-	+	+	+	+	-
Fels-2	N/A^†^		-	-	-	+	-	-
ST104	*artAB*	ADP-ribosyl transferase toxin homologue	-	-	-	-	-	+
CP4-57	N/A		-	-	+	-	-	-

*Prophage remnant.

^†^NA - Not applicable.

Prophages Gifsy-1, Gifsy-2 and the *sopE2* and *sspH2* phage remnants were present in all six genomes. They contain virulence genes commonly associated with type III effector proteins that are injected by the bacterium through type III secretion [41], and thus are important for virulence.

Fels-1 and Fels-2 were only found in strains, L847 (DT197) and L904 (DT9), respectively. The sparse presence of these prophages was not surprising as Fels-1 and Fels-2 are commonly absent in Typhimurium strains [42]. Fels-1 codes for virulence factors *nanH* and *sodCIII*[41] while Fels-2 harbours the gene *abiU* of unknown function [41]. ST104 prophage harbours *artAB* which codes for a putative toxin. It is often present in epidemic multiple drug resistant DT104 strains [43]. Interestingly, this prophage was also found in L927 (DT12a).

ST64B codes a virulence factor similar to Ssek NleB type III secreted effector proteins [44]. This prophage was present in four NGS strains, L847 (DT197), L852 (DT135a), L904 (DT9), and L945 (DT108), which were from both GC I and GC II. ST64B was also found in genome strains, SL1344 and 14028 S, but the prophage is defective in these strains due to a frameshift mutation in the open reading frame (ORF) SB21, which leads to the inability to produce infectious virions [45]. The same frameshift mutation in SB21 was found in L852 (DT135a) and L904 (DT9). Since SL1344, 14028 S, L852 (DT135a) and L904 (DT9) all belonged to GC II, the frameshift mutation may be an ancestral event shared by these GC II strains.

Prophage CP4-57 controls phage excision during the biofilm development stage which in turn enhances the motility in the host and increases biofilm dispersal while reducing growth [46]. This prophage has been found in *E. coli* strains suggesting a co-evolution between the two species [46]. This prophage was only found in strain L852 (DT135a). Since DT135a is a prevalent DT, further studies are warranted to determine the role of CP4-57 in adaptation and DT135a prevalence. Biofilm formation and dispersal are likely to be important for environmental survival leading to prevalence [47,48].

The P2-like phage SopEΦ was notably absent in all six strains sequenced in this study. SopEΦ contains the virulence gene *sopE*, and when disrupted reduces invasiveness [49]. This prophage was found in epidemic Typhimurium strains of DT204 and DT49 [39] and in two published genome strains SL1344 and ST4/74.

Altogether, the results suggest that prophages may not be maintained in all Typhimurium genomes. On the other hand, other studies have shown that prophages can be easily transferred between strains, particularly if the prophages are integrated at a tRNA site, like the case of ST104 and ST64B [50]. Thus, prophages make an important contribution to the diversification of Typhimurium genomes. The analysis also highlights the important roles prophages may play in virulence and potential adaptation of Typhimurium.

### Large indels

Regions of > 500 bp that are present in the reference genome LT2 but absent in the NGS strains are identified as large indels. Seven large indels were found in the six NGS strains with sizes ranging from 558 bp to 1,992 bp (Table 4). Indel events were cross-referenced with genomes SL1344, D23580, DT2 and 14028 S to see if they were carried on closely related isolates. Five indels were present in only one strain, with two indels shared in more than one strain. Deletion of STM2599 (*gipA*) was found in L927 (DT12a) and L847 (DT197) and the indel in STM0291 was found in L852 (DT135a) and SL1344. Three of the seven deletions encompassed a whole gene while the remaining four deletions were partial deletion of a gene leading to truncations of 15% to 37% of the gene. Truncations of more than 20% were treated as pseudogenes [51]. Thus, all, except STM0291 which only had a truncation of 15% of the gene, were likely to be nonfunctional. STM0291 is located in SPI-6 and codes for a RHS like protein. RHS family proteins have been known to be diverse [52]. Therefore, the deletion detected in L852 and SL1344 may be a variant of RHS.

**Table 4:** **Insertion and deletion locations relative to *S. enterica *serovar Typhimurium LT2 detected from the six genomes sequenced in this study**

**Genome (Phage type)**	**LT2 location**	**Deletion size (bp)**	**Affected gene**	**Gene function**	**Gene truncation (%)/Deletion**
L945 (DT108)	4,792,951-4,793,967	1,016	STM4534	Transcriptional regulator	Truncation (37)
L927 (DT12a)	2,905,570-2,907,184	1,614	STM2763, STM2764	Putative intergrase	Deletion
L927 (DT12a), L847 (DT197), DT104	2,748,190-2,749,475	1,285	STM2599 (*gipA*)	Gifsy-1 minor phage tail	Deletion
L852 (DT135a), SL1344	334,297-334,903	606	STM291	Putative RHS like protein	Truncation (15)
L847 (DT197)	975,166-977,158	1,992	STM905, STM906	Fels-1 hypothetical protein	Deletion
L847 (DT197)	341,423-342,445	1,022	STM299 (*safA*)	Putative outer membrane protein	Truncation (46)
L847 (DT197)	2,142,063-2,142,621	558	STM2066 (*sopA*)	Secreted effector protein	Truncation (24)

Interestingly, the genes deleted or truncated appeared to have a role in virulence. *gipA* is required for survival in Peyer’s patches [40]. *gipA* mutants have been shown to be attenuated to some extent after oral infection in mice, but displayed the same level of virulence as the wild type if inoculated intraperitoneally. *safA (*STM0299) is part of the *saf* fimbrial operon. *safA* mutants are attenuated in a pig model, but not in calves or chickens [53], and the same *saf* operon is not needed for virulence in mice [54,55]. *sopA* is used to alter host cell physiology and promote bacterial survival in host tissues [56]. *sopA* mutant has reduced *Salmonella*-induced polymorphonuclear leukocytes transepithelial migration [56].

Loss of these genes is expected to be disadvantageous to each corresponding strain, and may explain the differences in the ecology of several phage types. L847 (DT197) carries *gipA* deletion, *sapA* and *sopA* truncation. However, this phage type has increased in frequency in Australia in recent years, which argues against the importance of these genes for virulence in humans, although the increased detection of this phage type may be due to increased ability to colonise food animals, leading to increased exposure in humans. L927 (DT12a), also one of the most frequent phage types, carries *gipA* deletion and contains a truncated STM4534, a transcriptional regulator which regulates the phosphotransferase system and possibly other systems [57].

### Gene disrupting mutations

Apart from gene truncations due to partial deletion of a gene as described above, we identified 15 genes terminated earlier when compared to strain LT2, due to a stop codon (Table 5), leading to proteins that are >20% shorter and thus these genes were considered as pseudogenes. The distribution of these pseudogenes was mapped onto the SNP-based MP tree (data not shown). All, except one pseudogene (STM3745), was found only in a single strain as a single event. Earlier termination in STM3745 of unknown function resulted in 28% shorter protein and was observed in three strains of the same lineage, L796 (DT99), L904 (DT9) and D23580, suggesting a common ancestral event.

**Table 5:** List of genes affected by early stop codon

**Gene locus**	**Gene name**	**Gene function**	**COG**	**COG function**	Stop codon position*	**Gene size**	**Amino acid length**	**Protein length of original (%)**	**Genomes (Phage types)**
STM0054		Oxalacetate decarboxylase subunit beta	C	Energy production and conversion	766	1,302	434	59	DT2
STM0097	*polB*	DNA polymerase II	L	Replication, recombination and repair	1,726	2,352	784	73	L796 (DT99)
STM2261	*napF*	Ferredoxin-type protein	C	Energy production and conversion	157	492	164	32	L796 (DT99)
STM2315	*yfbK*	Hypothetical protein	R	General function prediction only	643	1,782	594	36	L927 (DT12a)
STM2517	*sinH*	Intimin-like protein			1,213	2,193	731	55	L927 (DT12a)
STM2758		Phosphotransferase system IIC component	G	Carbohydrate transport and metabolism	469	1,542	514	30	DT2
STM2771	*fljb*	Flagellin	N	Cell motility	337	1,521	507	22	T000240
STM3644	*bisC*	Biotin sulfoxide reductase	C	Energy production and conversion	148	2,334	778	6	L796 (DT99)
STM3666	*ysaA*	Oxidoreductase	C	Energy production and conversion	64	474	158	14	L796 (DT99)
STM3745		Cytoplasmic protein			613	852	284	72	L796 (DT99), L904 (DT9), D23580
STM4022	*yihT*	Aldolase	G	Carbohydrate transport and metabolism	430	879	293	49	L796 (DT99)
STM4339	*blc*	Outer membrane lipoprotein Blc	M	Cell wall/membrane/envelope biogenesis	127	534	178	24	L927 (DT12a)
STM4413		Metallo-dependent hydrolase	R	General function prediction only	382	1,164	388	33	L847 (DT197)
STM4495		Type II restriction enzyme methylase subunit	V	Defense mechanisms	334	1,092	364	31	L904 (DT9)
STM4593	*sthB*	Fimbrial usher protein	NU	Cell motility, intracellular trafficking and secretion	307	2,538	846	12	L796 (DT99)

*Based on Typhimurium genome strain LT2.

L796 (DT99) had the most number of strain specific pseudogenes followed by strains L927 (DT12a), L847 (DT197), DT2 and L904 (DT9) with seven, three, three, two and one pseudogenes, respectively. It is interesting to note that L796 (DT99) had a higher number of disrupted genes as well as the highest number of SNPs. This strain also had a 27% shorter DNA polymerase II encoded by *polB,* which may have resulted in a mutator phenotype.

Several pseudogenes for example, *napF* and *blc*, if active, are involved in energy conversion and metabolic pathways. *napF* encodes a predicted 3Fe-4S iron sulfur protein [58]. *NapF* mutant causes a growth defect under anaerobic conditions on glycerol/nitrate medium but is not essential for the activity of periplasmic nitrate reductase [59]. Therefore, NapF does not have a direct role in nitrate reduction but contributes to energy conservation. In *E. coli*, *blc* promoter is expressed during stationary growth phase which is controlled by *rpoS* sigma factor, directing the expression of genes necessary for adaptation to low nutrients condition. Therefore, both *napF* and *blc* are important for conserving energy.

Only one pseudogene, *sthB*, may be involved in host-restriction. *SthB*, if active, codes for a fimbrial usher protein. Deletion of *sthABCDE* operon results in reduced caecal colonisation in mice [60]. Furthermore, *sthB* mutants in chicken hosts have reduced colonisation [55]. This pseudogene was only found in L796 (DT99). Since this strain is only commonly associated with pigeons, this gene may have an effect on host restriction.

### Plasmid sequences

Most Typhimurium strains including LT2 carry a 90-kb virulence plasmid, pSLT [61]. It contains many known virulence genes including *spv* (*Salmonella* plasmid virulence), the *pef* (plasmid-encoded fimbriae) region, *rck* (resistance to complement killing), a homolog of *dsbA* (disulfide bond isomerase) and a homolog of the AraC family of transcriptional regulators [62-65]. The published genomes SL1344, DT2, D23580 and 14028 S were all found to contain pSLT. In order to determine the presence of pSLT from the 6 strains sequenced, reads and contigs were mapped onto the LT2 pSLT sequence (NC_003277). Reads homologous to pSLT were found in strains L796 (DT99), L847 (DT197), L852 (DT135a) and L904 (DT9) with 96%, 96%, 86% and 90% coverage to pSLT, respectively.

Strains L945 (DT108) and L927 (DT12a) contained reads covering only 2.6% and 0.8% of the pSLT plasmid suggesting that both of these strains did not have pSLT. It is likely that L945 (DT108) has lost the plasmid as all other strains from GC I contained the plasmid. In contrast, it is not known whether L927 (DT12a) has lost the plasmid or the plasmid was only gained after the divergence from the L927 (DT12a) lineage.

L945 (DT108) contained additional contigs that were not able to be aligned with LT2 chromosomal sequence or pSLT plasmid sequence. These contigs were then searched against GenBank using BLASTn. Eighty eight contigs, ranging from 104 bp to 5,980 bp, from L945 (DT108) had the closest match, with 65% DNA sequence identity, to the cryptic plasmid pHCM2 of 106 kb belonging to Typhi strain CT18. Sequence homologous to *repA* was identified in one of the contigs suggesting that a novel plasmid was present in L945 (DT108).

### Comparison of host adapted phage types: DT99 and DT2

Phage types of DT99 and DT2 are commonly associated with pigeons [3] and the mechanism for host-adaptation in these two phage types remains unknown. A previous microarray study on DT2 and DT99 phage types found that the loss of genetic regions does not correlate with host-adaptation [66]. The DT2 strain and L796 (DT99) were well separated within GC II and adaptation must have occurred independently. Comparison of their genomes did not identify any additional sequences that may contribute to host adaptation. There were few indels found common to both DT2 and L796 (DT99) that were not found in other genomes. A region between STM1555 to STM1559 was absent, which was previously reported [66]. This region encodes several proteins of putative functions including a transcriptional regulator; Na^+^/H^+^ antiporter; an aminotransferase; glycogen synthesis protein *glgX*[67] and glycosyl hydrolase. It is not clear whether the absence of this region is important for host-adaptation in pigeons since it is also absent in NCTC 13348 (DT104), a broad host strain. Other genetic elements commonly absent in both DT2 and L796 (DT99) were the Fels-1 and Fels-2 prophages. Again, both of these prophages were also absent in many of the other Typhimurium isolates.

Studies have shown that adaptation could be resulted from changes as small as one SNP, which can result in either increased or reduced virulence in animal models [68,69]. For example, an *rpoS* mutant LT2 strain has reduced virulence in mouse models [69]. Similarly, a nsSNP on *fimH* has been shown to improve the bacterial adhesion of serovar Enteritidis to chicken leukocytes [70]. There were no SNPs in either of these genes in strains L796 (DT99) and DT2. nsSNPs in *ycjF* were found in both L796 (DT99) and DT2, although the SNP locations differed between the two, at codons 301 and 324 for L796 (DT99) and codon 181 for DT2. *ycjF* codes for a hypothetical protein and its homolog in *E. coli* is essential for virulence *in vivo* in a mouse septicaemia model [71]. Other than that, there were no SNPs that were only found in both L796 (DT99) and DT2.

Comparative genome analysis of DT99 and DT2 revealed few genetic features that are specific to these two host adapted phage types. Therefore, multiple factors are likely to have contributed to the adaptation to pigeons.

## Conclusion

Six diverse Typhimurium strains based on our previous SNP typing study were investigated at the genome level and compared to seven other publicly available genomes to determine genetic variations that may contribute to their prevalence and host-adaptations. Variations between these genomes largely resulted from accumulation of SNPs. These genome-wide SNPs were also used to establish the phylogenetic relationships of 13 genome strains. Despite the presence of parallel/reverse mutations, the resulting phylogeny was generally consistent with our previous SNP typing study [6]. Other variations included prophages, plasmids and IS elements. The pSLT virulence plasmid was detected in all except two strains, L927 (DT12a) and L945 (DT108). Interestingly, L927 (DT12a) contained a novel plasmid with some similarities to cryptic plasmid pHCM2 first reported in Typhi CT18.

There was evidence of genome degradation, including pseudogene formation and some large indels. The pseudogenes mainly resulted from earlier termination codons or IS*200* insertions which appeared mostly to be random. However, some IS*200* insertions may provide a selective advantage including insertions in *icdA*, *accA* and *dacB*, all of which are related to antibiotic resistance.

Comparison of two host-adapted Typhimurium phage types, L796 (DT99) and DT2, did not reveal any unique genetic elements between them. SNP-based phylogeny grouped these strains together in GC II but they were clearly of separate lineages. This suggests that host-adaptation is a result of convergent evolution. However, factors contributing to the prevalence and host-adaptation in Typhimurium remain to be uncovered. In conclusion, genetic diversity within Typhimurium is mainly due to accumulation of SNPs, some of which led to pseudogenes. Unique genetic elements that were common between host-adapted phage types were not found.

## Competing interests

The authors declare that they have no competing interests.

## Authors’ contributions

Experimental work and data collection were carried out by SP. SP, SO, RL, LF, BL and PRR contributed to data analysis and interpretation. The study was conceived and designed by RL and LW. The manuscript was drafted by SP, SO and RL. All authors have read and approved the final manuscript.
